# Heat Shock Protein 70 Neutralizes Apoptosis-Inducing Factor

**DOI:** 10.1100/tsw.2001.322

**Published:** 2001-10-24

**Authors:** Guido Kroemer

**Affiliations:** Centre National de la Recherche Scientifique, UMR, Institut Gustave Roussy, Villejuif, France

**Keywords:** apoptosis, mitochondria, stress response

## Abstract

Programmed cell death (apoptosis) is the physiological process responsible for the demise of superfluous, aged, damaged, mutated, and ectopic cells. Its normal function is essential both for embryonic development and for maintenance of adult tissue homeostasis. Deficient apoptosis participates in cancerogenesis, whereas excessive apoptosis leads to unwarranted cell loss accounting for disparate diseases including neurodegeneration and AIDS. One critical step in the process of apoptosis consists in the permeabilization of mitochondrial membranes, leading to the release of proteins which normally are secluded behind the outer mitochondrial membrane[1]. For example, cytochrome c, which is normally confined to the mitochondrial intermembrane space, is liberated from mitochondria and interacts with a cytosolic protein, Apaf-1, causing its oligomerization and constitution of the so-called apoptosome, a protein complex which activates a specific class of cysteine proteases, the caspases[2]. Another example concerns the so-called apoptosis-inducing factor (AIF), another mitochondrial intermembrane protein which can translocate to the nucleus where it induces chromatin condensation and DNA fragmentation[3].

A recent article published in *Nature Cell Biology* by Ravagnan et al.[4] shows that heat shock protein (HSP) 70 can bind to AIF and neutralize its function. HSP70 is a major cytoprotective protein, meaning that its overexpression increases the resistance of cells to apoptosis induction. Its overexpression in transgenic animals suffices to cause cancer development[5], and several studies have shown a correlation with enhanced expression of HSP70 in tumor cells and poor responses to chemotherapy[6,7]. Elucidation of the mechanism of how HSP70 prevents apoptosis thus may furnish important clues to anti-cancer chemotherapy.

Many researchers working on the biochemical link between mitochondrial membrane permeabilization and lethal cellular catabolism have been concentrating on the role of caspases. Indeed, two proteins released from mitochondria can contribute to the activation of caspases, namely cytochrome *c* (via the activation of the apoptosome)[2] and Smac/DIABLO (via the neutralization of a caspase inhibitor, IAP)[8]. However, the knock-out of genes coding for essential apoptosome components (in particular Apaf-1 and pro-caspase-9) does not entail a complete abolition of developmental cell death during murine development (mice are born alive, though with major malformations, in particular in the central nervous system), indicating the existence of caspase-independent cell death pathways[9,10]. The knock-out of the AIF gene, however, appears to be incompatible with normal embryonic development and abolishes the first wave of (Apaf-1– and caspase-9–independent) morphogenetic cell death within the primitive ectoderm[11]. Considering that ontogeny recapitulates phylogeny, this appears a plausible result. Indeed, the fungus *Dictiostelium discoiderum* (which lacks caspases) has been recently found to possess an AIF-like protein that undergoes a mitochondrio-nuclear translocation during programmed cell death[12]. Moreover, the ancestors of AIF (but not that of caspases) can be found in bacteria[13]. AIF is a flavoprotein whose most conserved residues are those involved in the binding to its prosthetic group, flavine adenine nucleotide (FAD) and to its substrate nicotine adenine nucleotide (NAD). However, the NADH oxidase activity of AIF is not required for its apoptogenic function[14].

Heat shock proteins (HSP), particularly those of the HSP70 family, are also phylogenetically old molecules, with functional homologs in primitive bacteria. Under normal conditions, HSP70 proteins function as ATP-dependent molecular chaperones by assisting the folding of newly synthesized polypeptides, the assembly of multi-protein complexes, and the transport of proteins across cellular membranes. Under various stress conditions the synthesis of stress-inducible HSP70 enhances the ability of cells to cope with increased concentrations of unfolded or denatured proteins. HSP70 has also been reported to block apoptosis by binding Apaf-1, thereby preventing the constitution of the apoptosome[15,16]. As shown by Ravagnan et al.[4], however, overexpression of HSP70 can protect Apaf-1$^{-/-}$ cells against death induced by serum withdrawal, indicating that Apaf-1 is not the sole target of the anti-apoptotic action of HSP70. Indeed, proteins from the HSP70 family (but not those from other HSP families) were found to specifically interact with AIF. In a cell-free system, HSP70 prevents the AIF-induced chromatin condensation of purified nuclei. Cells overexpressing HSP70 are protected against the apoptogenic effects of AIF targeted to the extramitochondrial compartment. In contrast, an anti-sense HSP70 cDNA, which reduces the expression of endogenous HSP70, sensitizes to the lethal effect of AIF. The ATP binding domain of HSP70 is dispensable for AIF binding and AIF inhibition, although it is required for Apaf-1 binding. Such an HSP70 deletion mutant lacking the ATP binding domain is still effective in inhibiting cell death induced by serum withdrawal, menadione, and staurosporine, indicating that the chaperone function of Hsp70 (which relies on ATP binding and hydrolysis) is not essential for its AIF-inhibitory effect.

Altogether, these findings indicate that two phylogenetically old proteins, AIF and HSP70, exhibit mutual antagonism in cell death control. Intriguingly, the apoptosis-modulatory function of these two proteins are independent from their known enzymatic functions. More importantly, these data may have implications for drug design. On theoretical grounds, breaking the AIF-HSP70 interaction should sensitize HSP70 overexpressing cells to apoptosis induction. Moreover, it will be interesting to understand how the physical AIF-HSP70 interaction abolishes the pro-apoptotic function of AIF. Does HSP70 prevent AIF from reaching its target organelles, in particular the nucleus? Or do HSP70 and yet-to-be-identified positive mediators of apoptosis compete for the same binding site within AIF?
